# Analysis of prognostic factors of more/equal to10 years of survival for liver cancer patients after liver transplantation

**DOI:** 10.1007/s00432-018-2756-8

**Published:** 2018-09-26

**Authors:** Xinyu Li, Lei Huang, Xisheng Leng

**Affiliations:** Department of Hepatobiliary Surgery, Peking University People’s Hospital, Beijing, 100044 China

**Keywords:** Liver transplantation, Carcinoma, Hepatocellular, Survival rate, Risk factors, Prognosis

## Abstract

**Objective:**

To investigate prognostic factors of  more than 10 years of survival for liver cancer patients after liver transplantation.

**Methods:**

From May 2000 to May 2007, a total of 134 liver cancer patients who underwent liver transplantation in the Department of Hepatobiliary Surgery, Peking University People’s Hospital, were continuously and retrospectively enrolled. The patients included 120 males and 14 females. There were 124 cases (92.5%) of primary hepatocellular carcinoma, 9 cases (6.7%) of cholangiocarcinoma, and 1 case of mixed hepatocellular carcinoma and cholangiocarcinoma. Patients with perioperative death were excluded. Follow-up was performed until May 31st, 2017 or the time of death. According to the data on postoperative survival time, patients were divided into a < 10 years group (81 cases) and a ≥ 10 years group (53 cases). Patients’ clinical data were recorded and analyzed, including alpha-fetoprotein (AFP) level (≥ 400 µg/L or < 400 µg/L), number of tumor lesions (< 3 or ≥ 3), tumor size (≤ 5 cm or > 5 cm), vascular tumor thrombus (large blood vessel or non-large blood vessel), and histological differentiation degree. The Kaplan–Meier method was used to calculate survival rates. The log-rank method was used to compare the differences between survival curves. The Cox proportional hazards regression model was used to perform multivariate analyses of possibly influential factors.

**Results:**

(1) Follow-up was conducted with all 134 liver cancer patients after liver transplantation. The follow-up periods were 1–201 months, with a median of 18 (8.75, 132.5) months. The Kaplan–Meier survival analysis results showed that the 1-year, 3-year, 5-year, and 10-year cumulative survival rates were 70.3%, 48.6%, 46.8%, and 46.8%, respectively. (2) The differences in the age of patients, the incidence rate of AFP ≥ 400 µg/L, tumor histological differentiation, vascular tumor thrombi, tumor lesion size, and number of tumor lesions between two groups were all statistically significant (all *P* < 0.01). (3) The cumulative survival rates were different in AFP (log-rank *χ*^2^ = 13.428), histopathologic differentiation (log-rank *χ*^2^ = 33.592), large blood vessel tumor thrombi (log-rank *χ*^2^ = 36.470), tumor lesion size (log-rank *χ*^2^ = 39.835), and number of tumor lesions (log-rank *χ*^2^ = 47.016), and there were statistically significant differences between groups (all *P* < 0.01). (4) Multivariate Cox proportional hazards regression analyses showed that ≥ 3 tumor lesions [hazard ratio (HR) = 2.879, 95% confidence interval (CI) 1.566–5.422], tumor lesion size > 5 cm (HR = 2.682, 95% CI 1.382–5.366), large blood vessel tumor thrombi (HR = 1.831, 95% CI 1.010–3.341), and poor histological differentiation (HR = 2.150, 95% CI 1.372–3.394), were risk factors affecting the 10-year survival of liver cancer patients after liver transplantation (all *P* < 0.05).

**Conclusion:**

Tumor size, tumor number, large blood vessel tumor thrombi, and low tumor differentiation were all found to be independent risk factors affecting the 10-year survival rate after liver transplantation in liver cancer patients.

## Introduction

Primary liver cancer (abbreviated here as liver cancer) ranks 3rd in prevalence and 2nd in mortality among cancers in China (Fitzmaurice et al. 2015). Liver cancer is a common malignancy that severely threatens individual health. For early-stage liver cancer patients, hepatectomy, liver transplantation, and radiofrequency ablation therapy all provide high cure rates (Lencioni and Crocetti 2012; Doyle et al. 2012; Wedd et al. 2015; Forner et al. 2012). However, most liver cancer patients in China are already in the middle and late stages of the disease when they seek treatment; therefore, irrespective of whether these patients are treated with hepatectomies or radiofrequency ablation therapy, their recurrence rates of liver cancer are higher than those of patients with early-stage liver cancer. Liver transplantation can cure tumors and potential liver cirrhosis simultaneously and is not affected by reduced liver function; therefore, it is one of the most effective treatment methods for end-stage liver diseases (Forner et al. 2012; Adam et al. 2012). The tumor recurrence rates of Chinese liver cancer patients 1, 3, and 5 years after receiving liver transplants are 19.28%, 29.53%, and 33.69%, respectively. Reports from other countries show that the tumor recurrence rates of liver patients after liver transplantation are 8–20%. Thus, predicting risk factors for the long-term survival of liver cancer patients after liver transplantation is an important area of research (Wang et al. 2013; Bhoori and Mazzaferro 2014). Our center performed the first liver transplant for liver cancer in May 2000. With an increasing number of surgery cases, we have accumulated a considerable amount of data on patients who have survived for 10 years after surgery and have completed the follow-up period. This study aimed to investigate the prognostic factors of more/equal to 10 years of survival for liver cancer patients after liver transplantation to provide references for clinical diagnosis and treatment in liver transplantation surgeries, including standards for recipient selection, survival assessment, and tumor recurrence.

## Subjects and methods

### Subjects

After perioperative death cases were excluded, a total of 134 liver cancer patients who had undergone liver transplantation in the Department of Hepatobiliary Surgery, People’s Hospital, Peking University between May 2000 and May 2007 were continuously and retrospectively enrolled. The patients included 120 males and 14 females. The patients’ ages were 17–69 years. There were 124 cases (92.5%) of primary hepatocellular carcinoma, 9 cases (6.7%) of cholangiocarcinoma, and 1 case of mixed hepatocellular carcinoma and cholangiocarcinoma. The orthotopic liver transplantation and the piggyback liver transplantation were 79 cases and 55 cases, respectively. The waiting time for liver transplantation was 1–120 days, and the median waiting time was 31 days. Patients were divided into a < 10 years of survival group (81 cases) and a ≥ 10 years of survival group (53 cases) based on postoperative survival time. Inclusion criteria: liver transplantation surgery was performed by our center; diagnoses of primary hepatocellular carcinoma, cholangiocarcinoma, and mixed hepatocellular carcinoma and cholangiocarcinoma were all confirmed by surgical pathology; and patients finished follow-up and had complete data.

### Postoperative treatment

#### Immunosuppressive agents

After surgery, patients conventionally receive triple immunosuppressive therapy with calcineurin inhibitors (CNI), mycophenolate mofetil (MMF), and prednisone acetate. CNI include cyclosporine and tacrolimus (trade name: ‎Prograf). Patients who received cyclosporine before 2003 all switched to tacrolimus afterward. After 2003, all patients received tacrolimus. The target concentrations (valley values) at different treatment time points are shown in Table 1. MMF was administered orally at 1 g/day and was stopped 6 months postoperative. If the white blood cell count measured < 3 × 10^12^/L during this period, the dose was reduced to 0.5 g/day, or the drug was stopped. Without an acute rejection reaction, prednisone acetate treatment should last no more than 1 month.

**Table 1:** Target concentrations of tacrolimus and cyclosporine at different time periods after liver transplantation in liver cancer patients (µg/L)

Inhibitor name	Target concentration (valley value)				
	< 1 month	1–3 months	> 3 to 6 months	> 6 to 12 months	> 12 months
Tacrolimus	10–12	8–10	6–10	6–10	3–5
Cyclosporine	400–500	300–400	200–300	150–200	100–150

#### Antiviral treatment for hepatitis B virus (HBV)-related liver transplantation

Before nucleos(t)ide drugs were approved for clinical use, the regimen used for the prevention of HBV re-infection was based on lamivudine mixed with small doses of hepatitis B immunoglobulin. After nucleos(t)ide drugs were approved for clinical use, a regimen of nucleos(t)ide drugs mixed with small doses of hepatitis B immunoglobulin was used (Gu and Wang 2002; Chinese Society of Organ Transplantation, Chinese Medical Association; Chinese Society of Hepatology, Chinese Medical Association 2016). During HBV-related liver transplantation, an intravenous injection of 2000 IU hepatitis B immunoglobulin is given in the anhepatic phase to neutralize HBsAg in the recipient’s blood. Within 6 months postoperative of an HBV-related liver transplantation, HBsAg, HBV DNA, and anti-HBs titers are monitored to confirm the dose and frequency of hepatitis B immunoglobulin. After surgery, the valley value of the anti-HBs titer increased to 1000 IU/L within 1 week, and titers were greater than or equal to 500 IU/L within 3 months, greater than or equal to 200 IU/L between 3 and 6 months, and greater than or equal to 100 IU/L after 6 months. 6 months after liver transplantation, the anti-HBs titer, HBsAg, and HBV DNA levels were assessed once every 3 months (Chinese Society of Organ Transplantation, Chinese Medical Association; Chinese Society of Hepatology, Chinese Medical Association 2016).

### Follow-up method

Postoperative follow-ups were performed regularly. Within 2 months of discharge from the hospital, assessments were performed once per week. After 2 months, assessments were performed once per month. After 1 year, assessments were performed once every 3 months. During this period, if variations or other conditions occurred, the frequency of assessment was increased. Follow-up included routine blood tests, liver and kidney function tests, blood biochemical tests, measurement of the concentration of tacrolimus in the blood, serum hepatitis virology, measurement of alpha-fetoprotein (AFP) levels, and abdominal computed tomography (CT) [magnetic resonance imaging (MRI)]. Follow-ups were performed until May 31st 2017 or the time of death.

### Study methods

Patients’ clinical data were recorded and analyzed, including demographic data, etiology, smoking and drinking history (yes or no), antiviral history (yes or no), transcatheter arterial chemoembolization (TACE) history (yes or no), Child-Pugh stage (A, B, and C), AFP level (≥ 400 µg/L or < 400 µg/L), number of tumor lesions (< 3 or ≥ 3), tumor size (≤ 5 cm or > 5 cm), vascular tumor thrombus (large blood vessel or non-large blood vessel), and degree of histological differentiation (high, moderate, and low differentiation). Variables in clinical data that had statistically significant results were subjected to analyses of survival risk. The etiological data collected included hepatitis B, hepatitis C, hepatitis B + hepatitis C, and others. Other conditions assessed were non-viral hepatitis and liver diseases such as alcoholic liver diseases, cholestatic liver diseases, autoimmune hepatitis, sclerosing cholangitis, drug-induced hepatitis, hepatolenticular degeneration, and congenital biliary atresia. The portal vein and/or vena cava tumor thrombi were defined as large blood vessel tumor thrombi, while small blood vessel tumor thrombi, lymph node invasion, and no tumor thrombus were defined as non-large blood vessel tumor thrombi.

### Statistical analyses

JMP 13.0 statistical software was used for statistical analyses. The normality of measurement data was assessed using the Shapiro–Wilk test. The homogeneity of variances was tested using the Levene method. Measurement data that conformed to a normal distribution were expressed as $\bar {x} \pm s$ and comparisons between groups were performed using an independent samples *t* test. Measurement data that did not conform to a normal distribution were expressed as *M* (*P*_25_, *P*_75_), and comparisons between groups were performed using the rank sum test. Count data were expressed as cases (%), and comparisons between groups were performed using the *χ*^2^ test. The Kaplan–Meier method was used to calculate survival rates and plot survival curves. The log-rank method (*χ*^2^ test or Fisher’s exact test) was used to compare differences among survival curves. The Cox proportional hazards regression model was used for multivariate analyses of the variables (*P* < 0.05) in single factor analysis. The data results were statistically significant with the difference of *P* value < 0.05.

## Results and discussion

### Postoperative survival conditions

Follow-up was conducted with all 134 liver cancer patients after liver transplantation. The follow-up time ranged from 1 to 201 months, with a median follow-up time of 18 (8.75, 132.5) months. The Kaplan–Meier survival analysis results showed that the 1-year, 3-year, 5-year, and 10-year cumulative survival rates were 70.3%, 48.6%, 46.8%, and 46.8%, respectively.

### Comparison of baseline data

The ages of patients in the < 10 years of survival group were lower than those of patients in the ≥ 10 years of survival group, and the incidence rate of AFP ≥ 400 µg/L in the < 10 years of survival group was higher than that in the ≥ 10 years of survival group; the differences in age and the incidence rate of AFP ≥ 400 µg/L between these two groups were both statistically significant (both *P* < 0.05). The differences in all other baseline data between these two groups were not statistically significant (all *P* > 0.05) (Table 2).

**Table 2:** Comparison of baseline data from liver cancer patients with different survival times after liver transplantation

Group	Number of cases	Age	Liver disease duration	Gender [case (%)]		Primary disease [case (%)]			
		(year, *x* ± *s*)	*M* (year, *P*_25_, *P*_75_)	Male	Female	Hepatitis B	Hepatitis C	Hepatitis B + hepatitis C	Others
< 10 years group	81	49 ± 10	11 (5, 20)	73 (90.1)	8 (9.9)	70 (86.4)	1 (1.2)	2 (2.5)	8 (9.9)
≥ 10 years group	53	52 ± 9	16 (10, 20)	47 (88.7)	6 (11.3)	46 (86.8)	3 (5.66)	1 (1.89)	3 (5.66)
Test value		1.907^a^	1.691^b^	0.071^c^		2.852^c^			
*P* value		0.029	0.091	0.790		0.415			

Group	Number of cases	TACE history	Anti-virus history	Smoking history	Drinking history	AFP ≥ 400 µg/L	Child-Pugh stage [case (%)]		
		Case (%)					A	B	C
< 10 years group	81	15 (18.5)	16 (19.8)	27 (33.3)	26 (32.1)	41 (50.6)	28 (34.6)	34 (42.0)	19 (23.5)
≥ 10 years group	53	10 (18.9)	5 (9.4)	17 (32.1)	20 (37.7)	13 (24.5)	22 (41.5)	20 (37.7)	11 (20.8)
Test value		0.003^c^	2.733^c^	0.023^c^	0.450^c^	9.357^c^	0.658^c^		
*P* value		0.960	0.098	0.879	0.503	0.002	0.720		

*TACE* transcatheter arterial chemoembolization, *AFP* alpha-fetoprotein

^a^ *t* value

^b^ *Z* value

^c^ *χ* ^2^ value

### Comparison of tumor characteristics

The differences in tumor histological differentiation, vascular tumor thrombi, tumor lesion size, and number of tumor lesions between the two groups were all statistically significant (*P* < 0.01 for all parameters). The difference in tumor types between the two groups was not statistically significant (*P* > 0.05) (Table 3).

**Table 3:** Comparison of tumor characteristics of liver cancer patients with different survival times after liver transplantation (case [%])

Group	Number of cases	Cancer type			Histological differentiation		
		Hepatocellular carcinoma	Cholangiocarcinoma	Mixed hepatocellular carcinoma and cholangiocarcinoma	High	Moderate	Low
< 10 years group	81	73 (90.1)	7 (8.6)	1 (1.2)	8 (9.9)	45 (55.6)	28 (34.6)
≥ 10 years group	53	51 (96.2)	2 (3.8)	0 (0.0)	23 (43.4)	26 (49.1)	4 (7.6)
*χ* ^2^ value		2.358			27.073		
*P* value		0.308			< 0.01		

Group	Number of cases	Vascular tumor thrombus		Tumor lesion size (cm)		Number of tumor lesions	
		Large blood vessel	Non-large blood vessel	≤ 5	> 5	< 3	≥ 3
< 10 years group	81	40 (49.4)	41 (50.6)	23 (28.4)	58 (71.6)	31 (38.3)	50 (61.7)
≥ 10 years group	53	5 (9.4)	48 (90.6)	42 (79.2)	11 (20.8)	46 (86.8)	7 (13.2)
*χ* ^2^ value		31.232		34.854		33.603	
*P* value		< 0.01		< 0.01		< 0.01	

### Results of univariate analyses

**Fig. 1:**
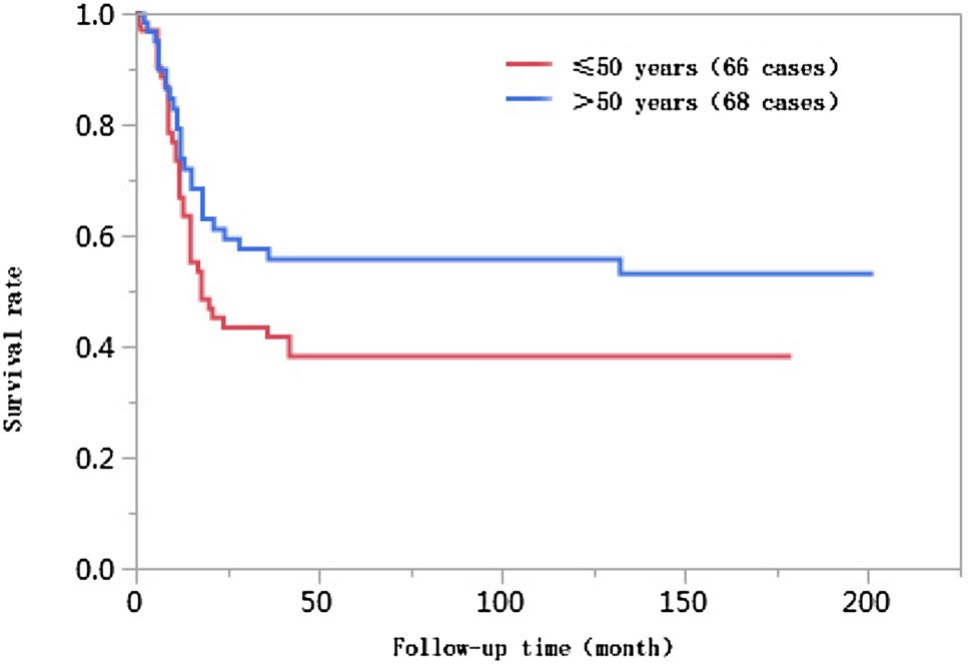
Comparison of survival curves of liver cancer patients at different ages after liver transplantation

**Fig. 2:**
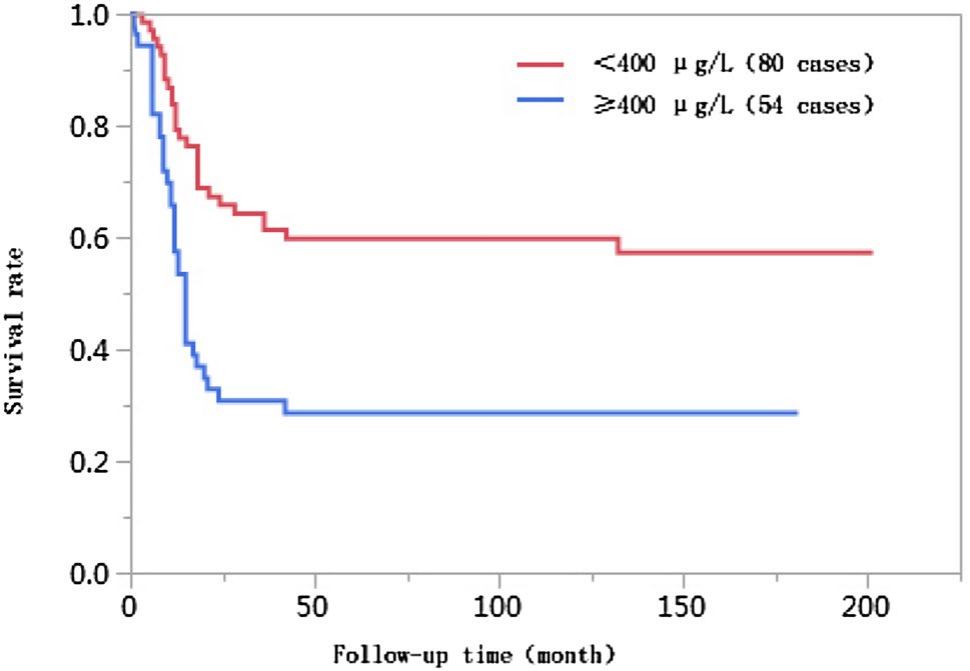
Comparison of survival curves of liver cancer patients with different AFP levels after liver transplantation

**Fig. 3:**
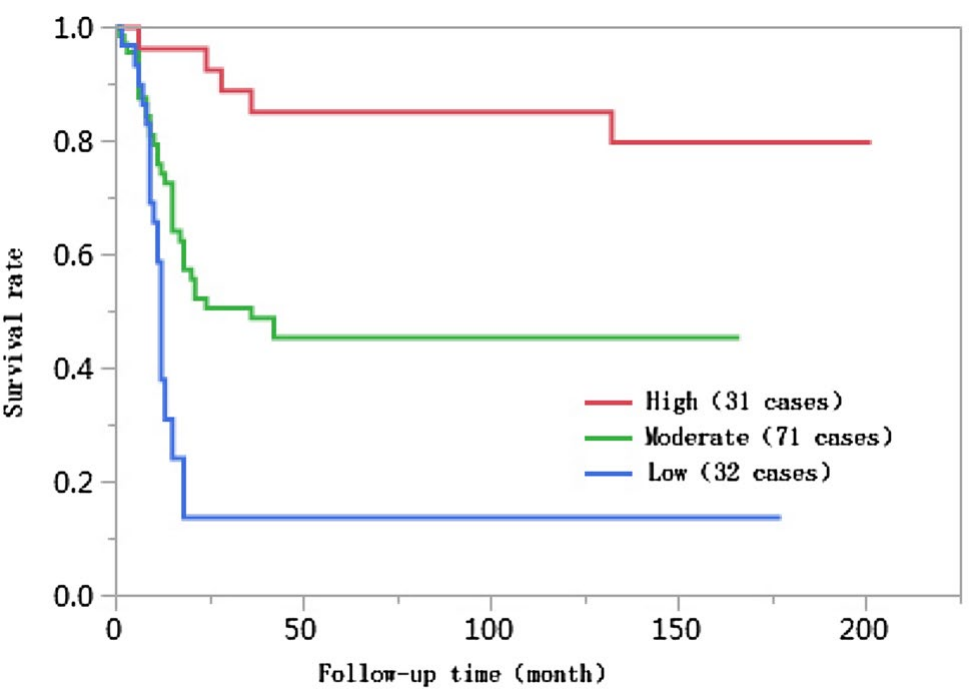
Comparison of survival curves of liver cancer patients with different degrees of histological differentiation after liver transplantation

**Fig. 4:**
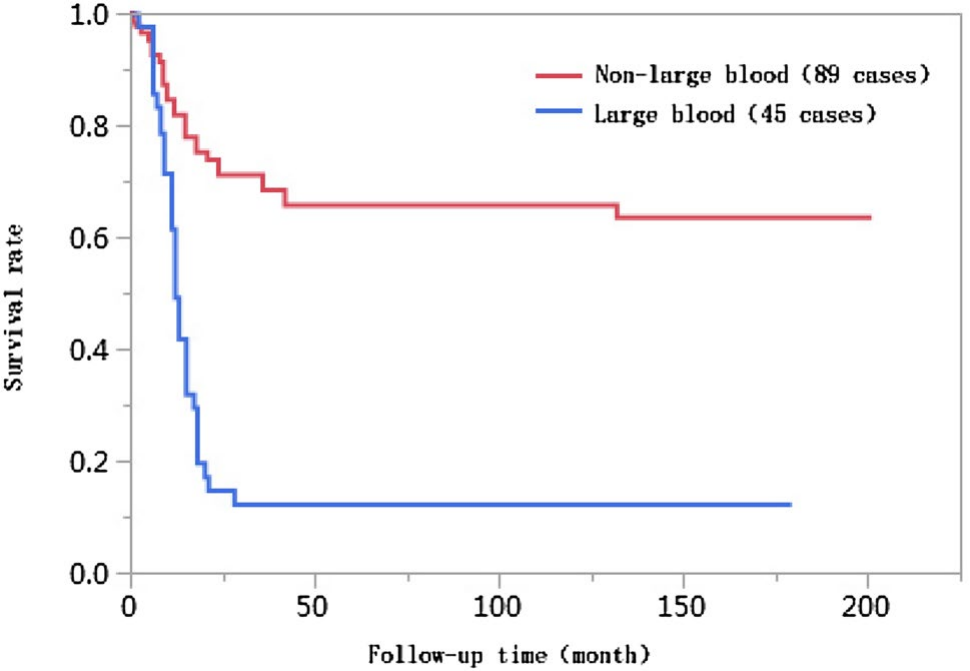
Comparison of survival curves of liver cancer patients with different vascular tumor thrombi after liver transplantation

**Fig. 5:**
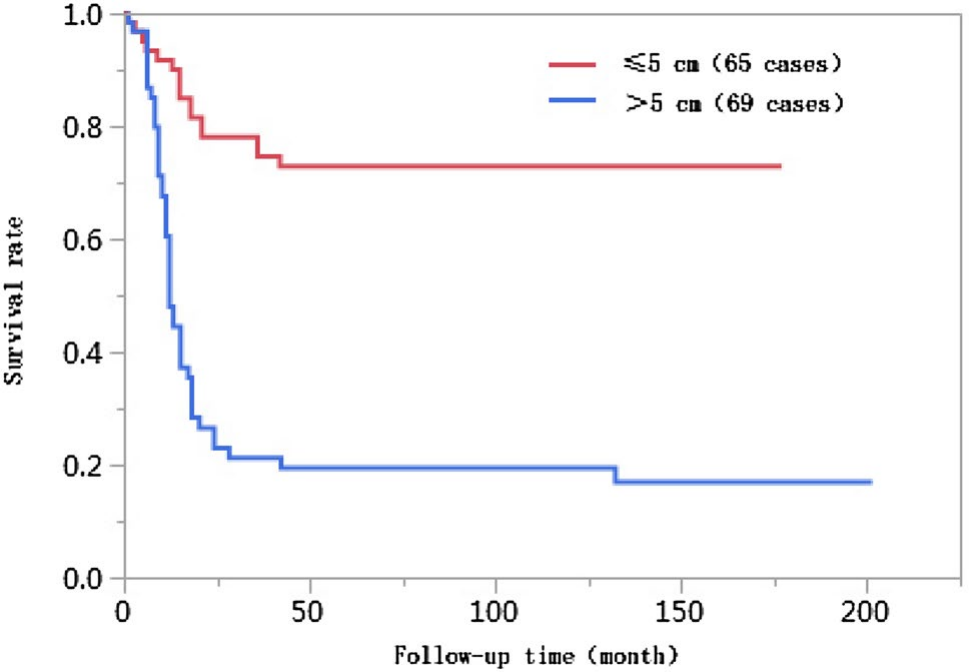
Comparison of survival curves of liver cancer patients with different tumor lesion sizes after liver transplantation

**Fig. 6:**
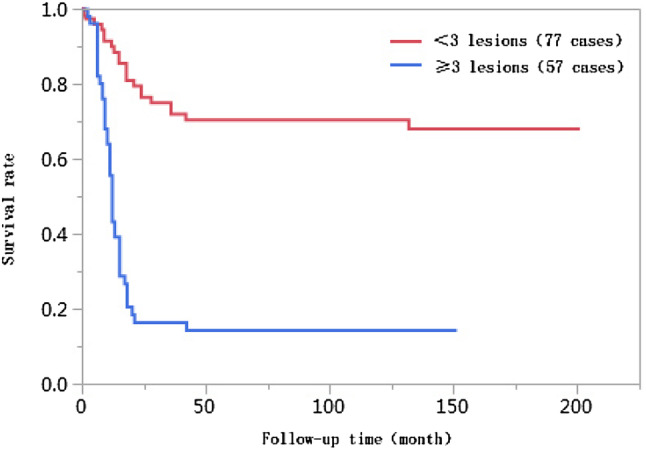
Comparison of survival curves of liver cancer patients with different numbers of tumor lesions after liver transplantation

The 1-year, 3-year, 5-year, and 10-year cumulative survival rates of patients at ages ≤ 50 were 66.8%, 41.7%, 38.2%, and 38.2%, respectively, and those of patients at ages > 50 years were 74.0%, 55.9%, 55.9%, and 55.9%, respectively. The differences in cumulative survival rates were not statistically significant (log-rank *χ*^2^ = 2.768, *P* = 0.096) (Fig. 1). The 1-year, 3-year, 5-year, and 10-year cumulative survival rates of patients with AFP < 400 µg/L were 79.5%, 61.5%, 62.0%, and 60.0%, respectively, and those of patients with AFP ≥ 400 µg/L were 57.4%, 30.8%, 28.6%, and 28.6%, respectively. The differences in cumulative survival rates were statistically significant (log-rank *χ*^2^ = 13.428, *P* = 0.0002) (Fig. 2). The 1-year, 3-year, 5-year, and 10-year cumulative survival rates of patients with high histopathological differentiation were 96.3%, 85.2%, 85.2%, and 85.2%, respectively, those of patients with moderate differentiation were 74.3%, 48.9%, 45.4%, and 45.4%, respectively, and those of patients with low differentiation were 38.1%, 13.8%, 13.8%, and 13.8%, respectively. The differences in cumulative survival rates were statistically significant (log-rank *χ*^2^ = 33.592, *P* < 0.01) (Fig. 3). The 1-year, 3-year, 5-year, and 10-year cumulative survival rates of patients with non-large blood vessel tumor thrombi were 81.9%, 68.4%, 65.6%, and 65.6%, respectively, and those of patients with large blood vessel tumor thrombi were 49.3%, 12.3%, 12.3%, and 12.3%, respectively. The differences in cumulative survival rates were statistically significant (log-rank *χ*^2^ = 36.470, *P* < 0.011) (Fig. 4). The 1-year, 3-year, 5-year, and 10-year cumulative survival rates of patients with tumor lesions ≤ 5 cm were 91.7%, 74.6%, 72.9%, and 72.9%, respectively, and those of patients with tumor lesions > 5 cm were 48.2%, 21.4%, 19.6%, and 19.6%, respectively. The differences in cumulative survival rates were statistically significant (log-rank *χ*^2^ = 39.835, *P* < 0.01) (Fig. 5). The 1-year, 3-year, 5-year, and 10-year cumulative survival rates of patients with < 3 tumor lesions were 89.9%, 71.9%, 70.4%, and 70.4%, respectively, and those of patients with ≥ 3 tumor lesions were 43.1%, 16.5%, 14.5%, and 14.5%, respectively. The differences in cumulative survival rates were statistically significant (log-rank *χ*^2^ = 47.016, *P* < 0.01) (Fig. 6).

### Multivariate analyses of factors that influenced the 10-year survival of liver cancer patients after liver transplantation

Multivariate Cox proportional hazards regression analyses indicated that ≥ 3 tumor lesions, tumor lesion size > 5 cm, large blood vessel tumor thrombi, and poor histological differentiation were associated with the 10-year survival of liver cancer patients after liver transplantation (all *P* < 0.05). Age and AFP level were not risk factors influencing the long-term survival of liver cancer patients after liver transplantation (*P* > 0.05 for both parameters) (Table 4).

**Table 4:** Results of Cox regression analyses on relevant prognostic factors of 10-year survival of liver cancer patients after liver transplantation

Factor	HR	95% CI	*P* value
Age	1.373	0.800–2.328	0.2465
AFP ≥ 400 µg/L	0.998	0.568–1.771	0.9955
Number of tumor lesions ≥ 3	2.879	1.566–5.422	0.0006
Tumor lesion size > 5 cm	2.682	1.382–5.366	0.0033
Large blood vessel tumor thrombus	1.831	1.010–3.341	0.0464
Histological differentiation	2.150	1.372–3.394	0.0008

*AFP* alpha-fetoprotein, *HR* hazard ratio, *95% CI* 95% confidence interval

### Discussion

Liver cancer is a highly prevalent cause of malignant tumors in China. Liver transplantation is an important therapeutic measure for this disease; studies in other countries show that the 5-year survival rate of liver cancer patients after liver transplantation can reach 63–80% (Silva et al. 2013; Agopian et al. 2015; Yao et al. 2015). There are few studies with large sample sizes and more than 10 years of follow-up on liver cancer patients after liver transplantation in China, and studies with large sample sizes and more/equal to 10 years of data are important for increasing the long-term survival of liver cancer patients after liver transplantation. Åberg et al. (2011) performed survival analysis studies on patients in Nordic countries 10 years after liver transplantation. The results showed that the 10-year survival rate was 66%. In this study, the 10-year cumulative survival rate of liver cancer patients after liver transplantation was 46.8%, which was slightly lower than the survival rates reported in other countries. This might be because all of the patients in this study were liver cancer patients. Liver cancer patients in European and American countries account for only 25–35% of the total number of liver transplants occurring every year (Byam et al. 2013). The other possible reason was that the patients in this study had been more serious. The 1-year, 3-year, 5-year, and 10-year cumulative survival rates of patients with non-large blood vessel tumor thrombi were 81.9%, 68.4%, 65.6%, and 65.6%, respectively, The 1-year, 3-year, 5-year, and 10-year cumulative survival rates of patients with tumor lesions ≤ 5 cm were 91.7%, 74.6%, 72.9%, and 72.9%, respectively, The 1-year, 3-year, 5-year, and 10-year cumulative survival rates of patients with < 3 tumor lesions were 89.9%, 71.9%, 70.4%, and 70.4%, respectively. In our study, this part of the data was equivalent to the results of European and American research centers, or even higher than their data (Silva et al. 2013; Agopian et al. 2015; Yao et al. 2015; Åberg et al. 2011).

Previous studies have shown that age is associated with the prognosis of liver cancer patients after liver transplantation (Wang et al. 2015). The results of this study show that the ages of patients in the < 10 years of survival group were lower than those in the ≥ 10 years of survival group [(49 ± 10) years vs. (52 ± 9) years, *P* < 0.05], suggesting that patients with survival times of less than 10 years were younger. The age of onset of malignant tumors in China tends toward younger ages (Chen 2016). Combined with the features of liver cancer etiology and epidemiology in China, the mean age of patients in these two groups was used as a cutoff value for the binary parameters ≤ 50 years and > 50 years. The Kaplan–Meier survival analysis results showed that the difference in cumulative survival rates between patients ≤ 50 years of age and patients > 50 years of age was not statistically significant (log-rank *χ*^2^ = 2.768, *P* = 0.096). The results of further Cox proportional hazards regression analyses showed that age was not associated with the 10-year survival of liver cancer patients after liver transplantation. Therefore, in this study, age was not an independent risk factor affecting postoperative long-term survival.

Alpha-fetoprotein is a tumor marker closely associated with liver cancer. A study by Yaprak et al. (2012) found that the preoperative AFP level was a relevant factor affecting survival. Different studies have proposed different AFP predictive values. Many studies use preoperative AFP > 400 µg/L as an independent risk factor of the prognosis after liver transplantation, and AFP > 400 µg/L has also been reported as a predictive factor of the prognosis of liver transplantation (Grąt et al. 2014; Zhang et al. 2014a, b; Hameed et al. 2014). The incidence rate of AFP ≥ 400 µg/L among patients in the < 10 years of survival group was significantly higher than that in the ≥ 10 years of survival group (*P* = 0.002). AFP was subjected to Kaplan–Meier survival analyses, and the results showed that the 1-year, 3-year, 5-year, and 10-year cumulative survival rates of patients with AFP ≥ 400 µg/L were all statistically significantly lower than those with AFP < 400 µg/L (log-rank *χ*^2^ = 13.428, *P* = 0.0002). These results suggest that, in a univariate analysis of the survival risk model, patients with AFP ≥ 400 µg/L have more severe diseases and poorer prognoses. However, the Cox proportional hazards regression analysis results showed that AFP ≥ 400 µg/L was not a risk factor affecting the long-term survival of liver cancer patients after liver transplantation. The survival analyses of Chen et al. (2010) on 109 liver cancer patients who underwent liver transplantation used AFP ≥ 400 µg/L as a cutoff value, and their results did not show that a high AFP level was a risk factor affecting tumor-free survival, which is similar to the result found in this study. There are several possible reasons for this result. First, in the univariate analyses, an increase in the AFP level was associated with long-term survival after transplantation. There may be synergistic effects of the AFP level and other risk factors, and the Cox proportional hazards model was used to correct for other factors; thus, an increase in the AFP level was no longer an independent risk factor for long-term survival. Some studies have used a cutoff value for AFP before liver transplantation of 1000 µg/L (Pawlik et al. 2005) and an AFP level ≥ 5000 µg/L (Zhang et al. 2015) as an independent predictive factor of poorer survival after liver transplantation. Therefore, defining the AFP cutoff as ≥ 400 µg/L may not be sufficient to predict risk. A study by Fawzy Montaser et al. (2012) reported that approximately 40% of early stage liver cancer patients have normal AFP levels; therefore, using AFP to predict tumor recurrence and metastasis after liver transplantation has limitations. Determining the predictive effect of AFP on survival risk requires further study.

Liver transplantation patients with preoperative vascular invasion had higher postoperative recurrence rates than those without vascular invasion and had poor postoperative prognoses (Lee et al. 2014). The 5-year survival rate after transplantation for patients with liver cancer combined with vascular invasion was 33.3%; however, for patients without vascular invasion, the survival rate was 68% (Hemming et al. 2001). The results in this study show that the incidence rate of large blood vessel tumor thrombi in the < 10 years of survival group (49.4%) was significantly higher than that in the ≥ 10 years of survival group (9.4%), and the difference in vascular tumor thrombi between these two groups was statistically significant (*P* < 0.01). The Kaplan–Meier survival analysis results showed that the 1-year, 3-year, 5-year, and 10-year cumulative survival rates of patients with non-large blood vessel tumor thrombi were significantly higher than those with large blood vessel tumor thrombi, and these differences were statistically significant (log-rank *χ*^2^ = 36.470, *P* < 0.01). Further inclusion of the results of multivariate Cox proportional hazards regression analyses suggested that a large blood vessel tumor thrombus was an independent risk factor affecting the long-term survival of patients after liver transplantation. The survival risk of patients with large blood vessel tumor thrombi was 1.83 times higher than that with non-large blood vessel tumor thrombi [95% confidence interval (CI) 1.010–3.341, *P* = 0.0464].

The importance of tumor size as a prognostic factor of liver cancer is undetermined. Tumor size has been reported as an important independent risk factor affecting the prognoses of liver cancer patients after liver transplantation; tumors with larger diameters more easily break through their capsules, and the postoperative recurrence rate is higher (Lee et al. 2014). When liver cancer is discovered later or due to the influences of the biological features of tumors, tumors with larger diameters increase the possibility of vascular invasion, causing tumor cells to enter the bloodstream. Other studies have reported that tumor size does not correlate with survival rate but is associated with factors of poor prognosis such as vascular invasion, low histological differentiation, and multiple lesions (Zhang et al. 2014). The percentage of patients with tumor diameters > 5 cm in the < 10 years of survival group was 71.6%, which was significantly higher than that in the ≥ 10 years of survival group (*P* < 0.01), while the 1-year, 3-year, 5-year, and 10-year cumulative survival rates of patients with tumor lesions ≤ 5 cm were higher than those of patients with tumor lesions > 5 cm (log-rank *χ*^2^ = 39.835, *P* < 0.01). Multivariate Cox proportional hazards regression analysis results indicated that tumor lesions with diameters > 5 cm were an independent risk factor affecting the long-term survival of patients after liver transplantation, and the risk of death for these patients was 2.68 times higher than that for patients with tumor lesions with diameters ≤ 5 cm (95% CI 1.382–5.366, *P* = 0.0033).

The number of tumors was a key factor affecting survival after liver transplantation (Lee et al. 2014). The condition of tumors, their number, and the distribution of tumors reflected the malignant biological behavior of invasive tumors. When more than two tumor lesions were present and tumor lesions were greater than a certain size, although the efficacy in the early stages after liver transplantation was better, increases in tumor metastasis and recurrence rates had a large influence on long-term survival (Zavaglia et al. 2005). The percentage of patients with ≥ 3 tumor lesions in the < 10 years of survival group was 61.7% in this study, which was significantly higher than that in the ≥ 10 years of survival group (13.2%), and this difference was statistically significant (*P* < 0.01). Studies on the association between the number of tumor lesions and the survival rate showed that the 1-year, 3-year, 5-year, and 10-year cumulative survival rates of patients with < 3 tumor lesions were higher than those with ≥ 3 tumor lesions, and this difference was statistically significant (log-rank *χ*^2^ = 47.016, *P* < 0.01). The multivariate Cox proportional hazards regression analysis results suggested that ≥ 3 tumor lesions was an independent risk factor affecting the long-term survival of liver cancer patients after liver transplantation, and the risk of death for patients with ≥ 3 tumor lesions was 2.879 times higher than that for patients with < 3 tumor lesions (95% CI 1.566–5.422, *P* = 0.0006).

Studies have suggested that the degree of histopathological differentiation of tumors is an independent risk factor affecting the prognoses of liver cancer patients after liver transplantation. When the histological differentiation of tumors is worse, the prognoses of liver transplantation recipients after surgery are poorer, and the survival times after liver transplantation of recipients with high and moderate degrees of differentiation are longer than those with low degrees of differentiation (Varona et al. 2015). Liver cancer cells with low degrees of differentiation are strongly invasive, grow rapidly, and easily break through the tumor capsule. The differences in the degree of histological differentiation between the two groups in this study were statistically significant (*χ*^2^ = 27.073, *P* < 0.01). The 1-year, 3-year, 5-year, and 10-year cumulative survival rates of patients with high degrees of differentiation were 96.3%, 85.2%, 85.2%, and 85.2%, respectively, those of patients with moderate degrees of differentiation were 74.3%, 48.9%, 45.4%, and 45.4%, respectively, and those of patients with low degrees of differentiation were 38.1%, 13.8%, 13.8%, and 13.8%, respectively. The differences in the cumulative survival rates were statistically significant (log-rank *χ*^2^ = 33.592, *P* < 0.01). With a reduction in the degree of differentiation, the risk of death for liver patients after liver transplantation increased 2.15 times (95% CI 1.372–3.394, *P* = 0.0008).

Univariate Kaplan–Meier survival curves showed that, when influenced by risk factors such as tumor size, tumor number, large blood vessel invasion, and lower tumor differentiation degree, the cumulative time of death for patients after liver transplantation was concentrated before 42 months, and survival curves were stable after 42 months. These results suggest that liver cancer recurrence and metastasis and resultant death primarily occur within 4 years of liver transplantation in liver cancer patients and might have smaller influences on the long-term survival of liver cancer patients more than 4 years after liver transplantation.

## Conclusions

In summary, tumor size, tumor number, large blood vessel tumor thrombi, and low tumor differentiation were independent risk factors for the 10-year survival of liver cancer patients after liver transplantation. However, because this study was a single-center retrospective analysis, the standards for liver transplantation indications, such as the selection of recipients, might differ from those used in other centers. The statistical data in this study were from the first 10 years of liver transplants for liver cancer patients performed at our center, and surgical techniques have undergone a gradual process of maturation; therefore, our study results require validation with multi-center studies with large sample sizes. In the future, we will further explore the long-term survival state of this group of patients.
